# Hyperglycemia potentiates a shift from apoptosis to RIP1-dependent necroptosis

**DOI:** 10.1038/s41420-018-0058-1

**Published:** 2018-05-10

**Authors:** William D. McCaig, Payal S. Patel, Sergey A. Sosunov, Nicole L. Shakerley, Tori A. Smiraglia, Miranda M. Craft, Katharine M. Walker, Matthew A. Deragon, Vadim S. Ten, Timothy J. LaRocca

**Affiliations:** 1 Department of Basic and Clinical Sciences, Albany College of Pharmacy and Health Sciences, Albany, NY 12208 USA; 2 Department of Pediatrics, Columbia University, New York, NY 10032 USA

## Abstract

Apoptosis and necroptosis are the primary modes of eukaryotic cell death, with apoptosis being non-inflammatory while necroptosis is highly inflammatory. We previously demonstrated that, once activated, necroptosis is enhanced by hyperglycemia in several cell types. Here, we determine if hyperglycemia affects apoptosis similarly. We show that hyperglycemia does not enhance extrinsic apoptosis but potentiates a shift to RIP1-dependent necroptosis. This is due to increased levels and activity of RIP1, RIP3, and MLKL, as well as decreased levels and activity of executioner caspases under hyperglycemic conditions following stimulation of apoptosis. Cell death under hyperglycemic conditions was classified as necroptosis via measurement of markers and involvement of RIP1, RIP3, and MLKL. The shift to necroptosis was driven by RIP1, as mutation of this gene using CRISPR–Cas9 caused cell death to revert to apoptosis under hyperglycemic conditions. The shift of apoptosis to necroptosis depended on glycolysis and production of mitochondrial ROS. Importantly, the shift in PCD was observed in primary human T cells. Levels of RIP1 and MLKL increased, while executioner caspases and PARP1 cleavage decreased, in cerebral tissue from hyperglycemic neonatal mice that underwent hypoxia-ischemia (HI) brain injury, suggesting that this cell death shift occurs *in vivo*. This is significant as it demonstrates a shift from non-inflammatory to inflammatory cell death which may explain the exacerbation of neonatal HI-brain injury during hyperglycemia. These results are distinct from our previous findings where hyperglycemia enhanced necroptosis under conditions where apoptosis was inhibited artificially. Here we demonstrate a shift from apoptosis to necroptosis under hyperglycemic conditions while both pathways are fully active. Therefore, while our previous work documented that intensity of necroptosis is responsive to glucose, this work sheds light on the molecular balance between apoptosis and necroptosis and identifies hyperglycemia as a condition that pushes cells to undergo necroptosis despite the initial activation of apoptosis.

## Introduction

Necroptosis is a programmed cell death (PCD) that occurs in cell types ranging from mature erythrocytes and leukocytes to neurons and cardiac cells^1–4^. This PCD results in a pro-inflammatory outcome^5–7^ due to loss of membrane integrity, cellular swelling, lysis, and release of cellular components^8,9^. Apoptosis, on the other hand, is characterized by cellular shrinkage, apoptotic body formation, and removal making it non-inflammatory^8,10,11^.

Although induction and signaling steps overlap, subsequent intracellular cascades diverge between the two forms of PCD^12^. Necroptosis occurs independent of caspases and instead is facilitated by receptor interacting protein kinase 1 (RIP1), receptor interacting protein kinase 3 (RIP3), and mixed lineage kinase domain-like (MLKL) protein^13,14^. These proteins comprise the necrosome complex^15^, triggered by a variety of extrinsic stimuli including tumor necrosis factor alpha (TNF-α), Fas ligand (FasL), and TNF-related apoptosis-inducing ligand (TRAIL)^16–19^. MLKL is phosphorylated downstream of RIP1 and RIP3 causing it to oligomerize and translocate to the plasma membrane where it forms pores^20–22^.

Downstream effects of necroptosis include stimulation of glycolysis and metabolism^5,23^ which is responsible for the production of toxic reactive oxygen species (ROS) and advanced glycation end-products (AGEs)^5,24,25^. ROS are generated in the mitochondria via the electron transport chain downstream of glucose metabolism, therefore, increased cellular glucose leads to increased production of ROS^26^. AGEs are produced from methylglyoxal, a direct byproduct of glycolysis^24,27^. Both ROS and AGEs induce cell damage, underscoring a central role for glucose in necroptosis^5^.

Previously, we showed that hyperglycemia increases necroptosis after it is activated in the context of artificial inhibition of apoptosis^23^. In that work we also demonstrated that hypoxia-ischemia (HI) brain injury in neonatal mice is exacerbated in hyperglycemia due to RIP1-dependent necroptosis^23^. In the present study, we tested the responsiveness of apoptosis to changes in cellular glucose. We found that while cell death is upregulated in high glucose following stimulation of extrinsic apoptosis, cell death is executed via necroptosis despite the specific stimulation of apoptosis. This indicates a distinct change in cell death signaling in normal and high glucose following the activation of apoptosis. Once apoptosis is activated, high glucose affects cell signaling such that apoptosis mechanisms cease shifting instead to necroptosis. We probe this novel shift in cell death mechanisms and explore the key molecules involved. This is significant as it reveals information about the molecular balance between apoptosis and necroptosis and is relevant to neonatal HI-brain injury.

## Results

### Hyperglycemia enhances cell death following induction of apoptosis, in a caspase-independent manner

**Fig. 1:**
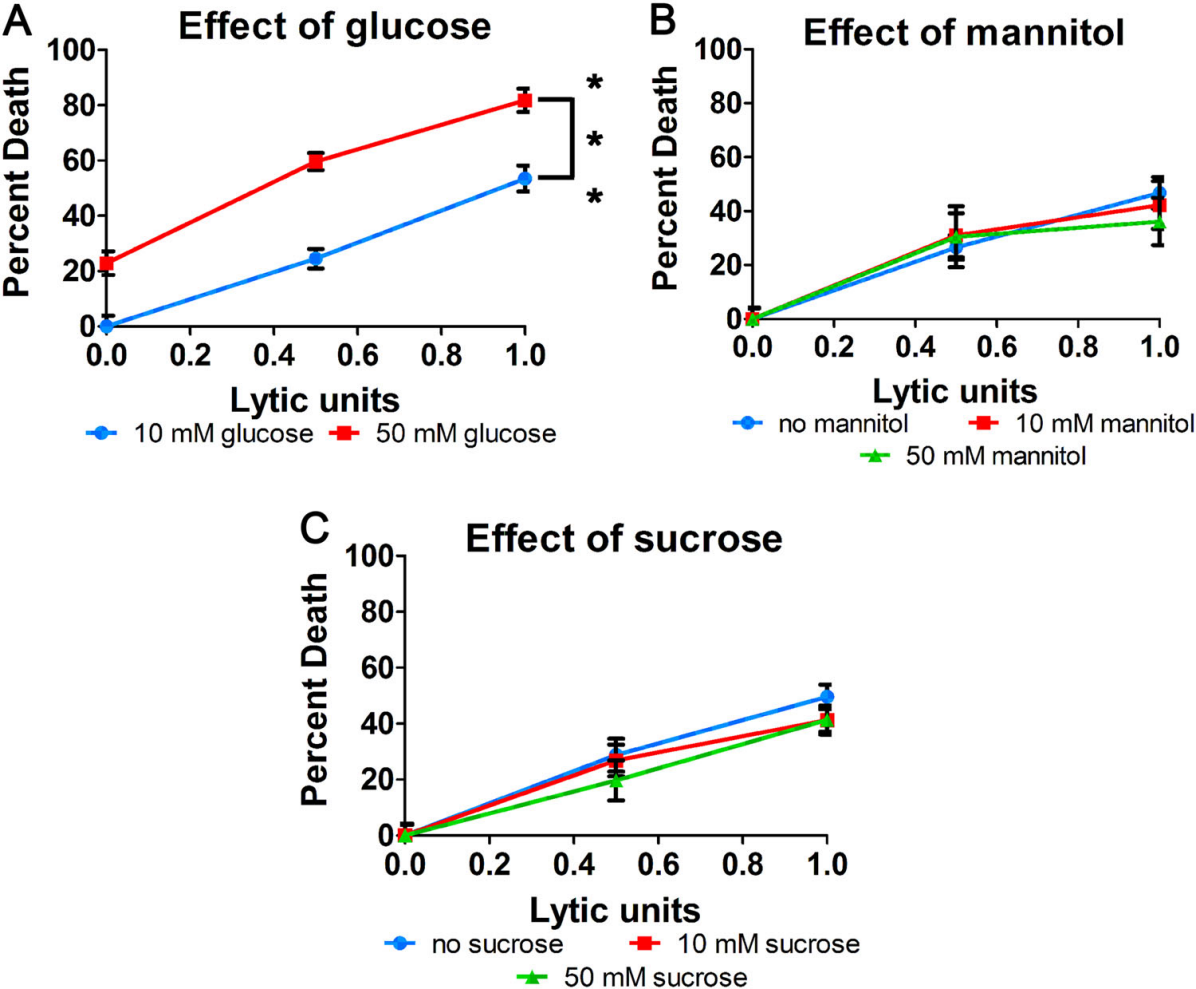
High glucose enhances cell death by the apoptotic stimulus, TNF-α/CHX, independent of osmotic pressure. **a** Cell death of U937 cells induced by the extrinsic apoptotic stimulus, TNF-α/CHX, is enhanced by high glucose. Enhancement of cell death does not occur in response to high levels of **b** mannitol or **c** sucrose. Two-way ANOVA, ***p < 0.001

To determine if extrinsic apoptosis is responsive to hyperglycemia we measured cell death of U937 monocytes in normal and high glucose conditions induced by TNF-α, a stimulus of extrinsic apoptosis. We chose 10 mM and 50 mM to represent normal and high glucose as U937 cells are maintained by passage in media containing 10 mM glucose. Thus, 50 mM glucose represents a 5-fold increase in glucose above normal for these cells, equivalent to critical hyperglycemic levels. Cell death following induction of apoptosis was enhanced in hyperglycemic conditions (Fig. 1a). To determine if the observed effect was due to osmotic imbalance, we replaced glucose with alternate sugars and measured cell death. The enhanced cell death following induction of apoptosis in high glucose was not observed with high levels of mannitol (Fig. 1b) or sucrose (Fig. 1c) suggesting that the increased cell death in high glucose is not due to osmotic effects. Overall, these results suggest that high glucose may upregulate extrinsic apoptosis.

For the experiments described above, and all others in this report, cyclohexamide (CHX) was administered with TNF-α, unless indicated otherwise. While the dose of CHX used throughout this report produces some cell death on its own (Fig. S1), CHX was included in all negative controls in this report, thus ruling out any effect of CHX alone. In addition, throughout this report the dose of TNF-α and FasL used in cell death assays was 0.4 lytic units (LD_50_) unless indicated otherwise. One lytic unit (LD_50_) is defined as the amount of TNF-α or FasL which produces 50% cell death (Fig. S2).

**Fig. 2:**
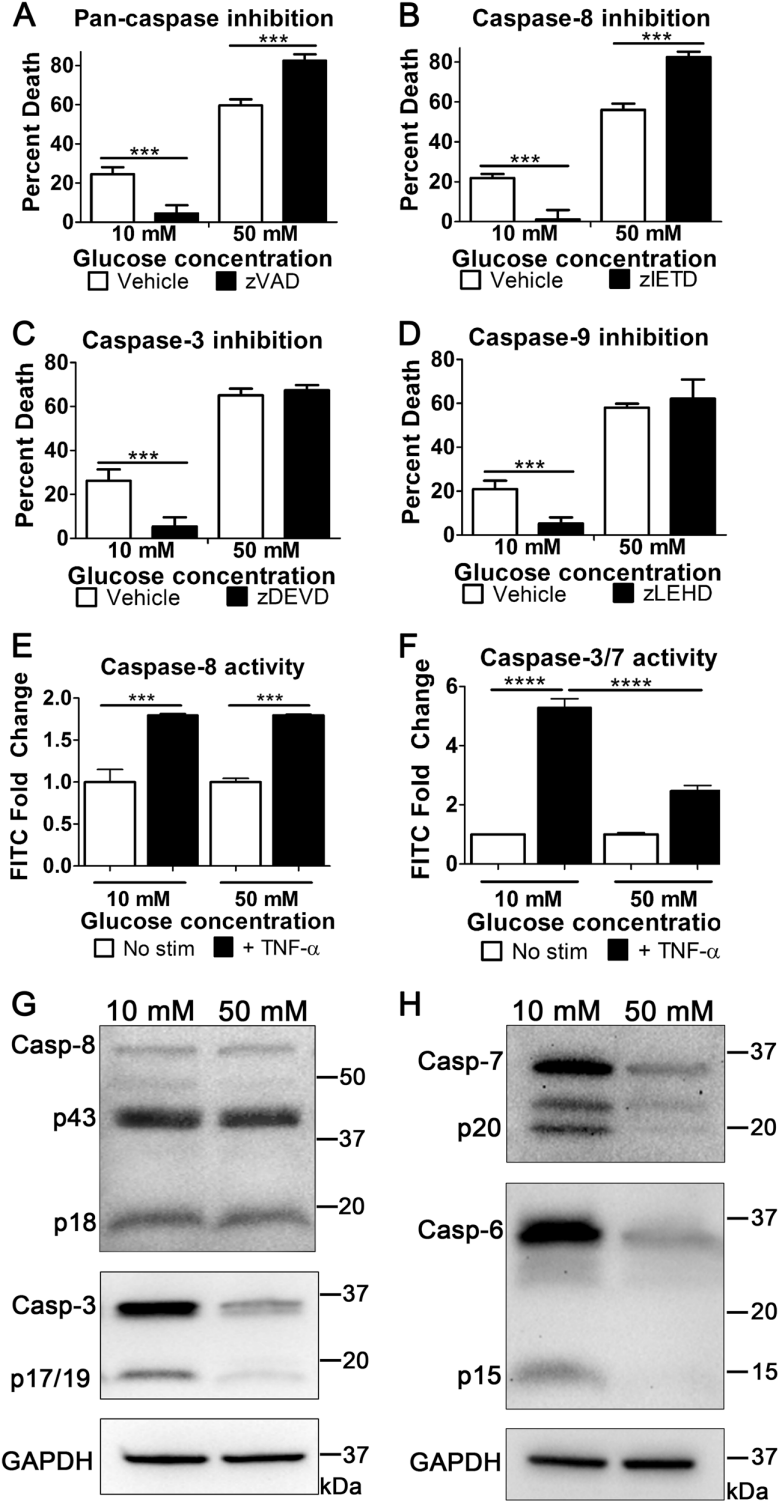
TNF-induced cell death proceeds in a caspase-independent manner in high glucose. **a** U937 cell death induced by the apoptotic stimulus, TNF-α/CHX, is prevented by the pan-caspase inhibitor, zVAD, at 10 mM glucose but not at 50 mM glucose. In addition, cell death at 50 mM glucose is not prevented by inhibition of **b** caspase-8 with zIETD, **c** caspase-3 with zDEVD, or **d** caspase-9 with zLEHD. FLICA flow cytometry measuring activation of **e** caspase-8 and **f** caspase-3/7 in normal and high glucose following stimulation with the apoptotic stimulus, TNF-α/CHX for 6 h at 37 °C and 5% CO_2_. Displayed is fold increase in FLICA staining in TNF-treated cells over untreated cells Activation of caspase-8 does not change in high glucose while activation of caspase-3 decreases. Two-way ANOVA, ***p < 0.001, ****p < 0.0001. **g** Western blots showing levels of caspase-8 and caspase-3 at normal and high glucose following stimulation with TNF-α/CHX. Caspase-8 levels do not change in high glucose while caspase-3 levels decrease. **h** Western blots showing levels of caspase-7 and caspase-6 at normal and high glucose following stimulation with TNF-α/CHX. Levels of both caspase-7 and caspase-6 decrease in high glucose

We next determined if the increased cell death at high glucose was due to enhanced caspase-dependent apoptosis. The pan-caspase inhibitor, zVAD-fmk, prevented TNF-induced cell death in normal glucose but not in high glucose conditions (Fig. 2a). Additionally, specific inhibitors for caspase-8 (initiator caspase for extrinsic apoptosis, Fig. 2b), caspase-3 (central executioner caspase, Fig. 2c), and caspase-9 (initiator caspase for intrinsic apoptosis, Fig. 2d) did not prevent cell death in high glucose. This indicates that high glucose increases TNF-induced cell death following induction of apoptosis in a caspase-independent manner.

### Levels and activity of executioner caspases decrease following TNF-α stimulation in high glucose

Due to the lack of caspase dependence in the hyperglycemic enhancement of cell death following induction of apoptosis, we wanted to determine the levels and activity of caspase-8 and executioner caspases-3, -6, and -7. FLICA caspase activity assay showed no change in caspase-8 activity (Fig. 2e) whereas caspase-3/7 activity decreased in high glucose (Fig. 2f). To determine the total caspase levels, we measured their abundance in cell lysates. Caspase-8 was unchanged in normal and high glucose conditions whereas levels of caspase-3, -6, and -7 decreased in high glucose following TNF-α treatment (Fig. 2g, h). In western blots that measured caspase-8 levels, full-length procaspase-8 was less abundant than its active cleavage products following TNF-α treatment (Fig. 2g). This is in contrast to the executioner caspases which were measured (Fig. 2g, h). To ensure that we identified procaspase-8 correctly, we performed western blots on lysates from U937 monocytes that were not treated with TNF-α. These results showed that procaspase-8 does indeed decrease in abundance following TNF-α treatment in contrast to procaspase-3 (Fig S3). Collectively, these results demonstrate that hyperglycemic conditions partially inhibit executioner caspases during TNF-induced cell death.

### Hyperglycemia causes a shift from extrinsic apoptosis to RIP1-dependent necroptosis

**Fig. 3:**
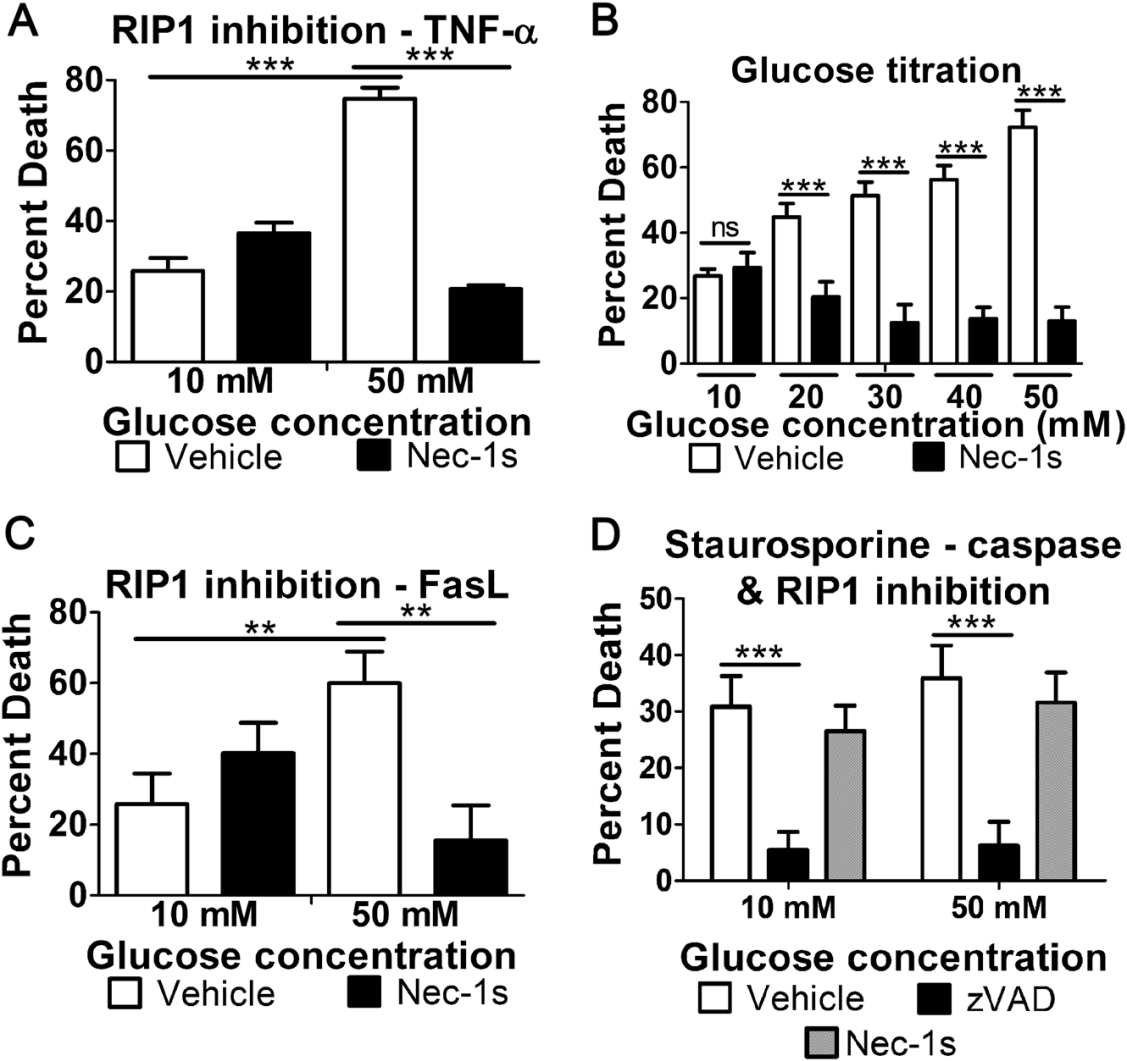
High glucose shifts extrinsic apoptosis to RIP1-dependent cell death. **a** Enhanced U937 cell death in response to the apoptotic stimulus, TNF-α/CHX, in high glucose conditions is prevented by the RIP1 inhibitor, nec-1s. **b** TNF/CHX-induced cell death of U937 cells depends on RIP1 across a range glucose concentrations between 20 and 50 mM. **c** Death of Jurkat T cells induced by FasL/CHX is enhanced in high glucose conditions in a RIP1-dependent manner. **d** Death of U937 cells by the intrinsic apoptotic stimulus, staurosporine/CHX, is prevented by pan-caspase inhibition with zVAD at 10 mM and 50 mM glucose. Inhibition of RIP1 with nec-1s has no effect on cell death at either glucose concentration. Two-way ANOVA, **p < 0.01, ***p < 0.001

The increase in cell death following induction of apoptosis in response to high glucose occurs in a caspase-independent manner (Fig. 2). This indicates that apoptosis does not increase in high glucose but, rather, changes to another cell death mechanism. Since necroptosis shares early steps with extrinsic apoptosis^10^, we wondered if high glucose causes a shift from extrinsic apoptosis to necroptosis. To test this hypothesis, we used the RIP1 inhibitor, nec-1s, to prevent necroptosis. We found that the hyperglycemic enhancement of cell death following induction of apoptosis was prevented by RIP1 inhibition (Fig. 3a). This suggests that high glucose potentiates a shift from extrinsic apoptosis to RIP1-dependent cell death. We performed similar death assays to show that several concentrations of glucose between 20 and 50 mM promote a shift to RIP1-dependent cell death (Fig. 3b). We found that FasL-induced cell death of Jurkat T cells increases in high glucose in a RIP1-dependent manner (Fig. 3c). This confirms that the hyperglycemic shift from apoptosis to RIP1-dependent necroptosis is shared between different cell types and different stimuli of cell death. Next, we asked if intrinsic apoptosis similarly shifts to necroptosis in high glucose conditions using staurosporine as a stimulus^28^. Intrinsic cell death by staurosporine (Fig. 3d) was not enhanced and remained caspase dependent in high glucose conditions. Therefore, the hyperglycemic shift to RIP1-dependent necroptosis is specific to extrinsic apoptosis.

**Fig. 4:**
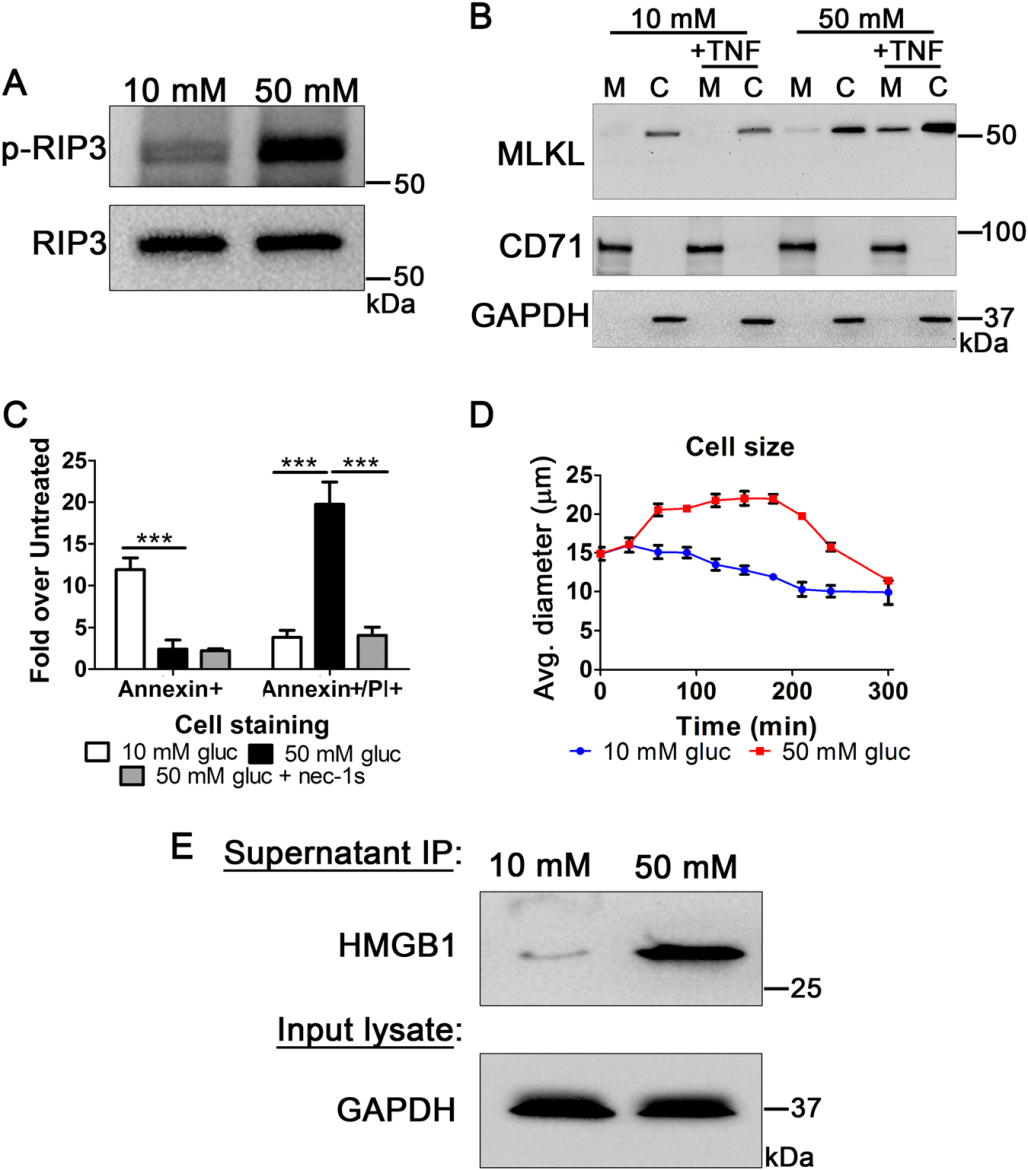
High glucose-induced shift to RIP1-dependent cell death is concurrent with increased markers of necroptosis and necrosis. **a** Levels of total RIP3 from U937 cells treated with TNF-α/CHX in 10 or 50 mM glucose were normalized to one another and phosphorylation of RIP3 was measured. RIP3 phosphorylation increases in high glucose conditions. **b** MLKL migrates to the membrane/organelle fraction (M) of U937 cells following TNF/CHX-induced death in high glucose conditions but not in normal glucose conditions. MLKL also increases in abundance in the cytoplasmic fraction (C) following TNF-induced death in high glucose conditions. CD71 = membrane-specific protein, GAPDH = cytoplasm-specific protein. **c** Flow cytometry of U937 cells following activation of TNF/CHX-induced apoptosis. Annexin V staining (apoptosis marker) decreases in high glucose following TNF/CHX treatment. Annexin/PI staining (necroptosis marker) increases in high glucose and is prevented by the RIP1 inhibitor, nec-1s. Two-way ANOVA, ***p < 0.001. **d** U937 cells shrink in diameter following TNF/CHX treatment in 10 mM glucose. At 50 mM glucose cellular diameter increases prior to cellular shrinkage. **e** Immunoprecipitation (IP) of HMGB1 from supernatants of U937 cells treated with TNF-α/CHX at low (10 mM) and high (50 mM) glucose. High levels of HMGB1 are released into the supernatant high glucose conditions

Due to the involvement of RIP3 and MLKL in necroptosis^14^, we wanted to determine if these factors played a role in the hyperglycemic shift to necroptosis. We found that RIP3 phosphorylation increases dramatically following TNF-α treatment in high glucose (Fig. 4a). Moreover, via cell fractionation we found that MLKL migrates to the membrane/organelle in high glucose conditions following TNF-α treatment (Fig. 4b). In addition, total levels of MLKL increase in the cytoplasm during the hyperglycemic shift to necroptosis. These results suggest a role for both RIP3 and MLKL in the hyperglycemic shift from apoptosis to necroptosis.

We also performed several experiments to characterize the cell death in hyperglycemia as necrotic in nature. First, we conducted flow cytometry analysis using cellular markers of apoptosis (annexin) and necroptosis (annexin/PI) in normal and high glucose conditions. Following TNF-α treatment, there was a decrease in annexin-positive apoptotic cells which was coincident with an increase in annexin/PI-double positive necroptotic cells in high glucose conditions (Fig. 4c). The increase in annexin/PI-double positive cells was inhibited by nec-1s, indicating RIP1 dependence on the increase in this necroptotic marker (Fig. 4c). Next, we analyzed cell size following TNF-α treatment in normal and high glucose conditions. We observed a steady increase in cell size in high glucose conditions followed by a steep decrease (Fig. 4d). This reflects a key morphological change that occurs to necroptotic cells as these cells swell initially followed by cell shrinkage due to lysis and expulsion of cellular contents^7,29^. We also observed the release of HMGB1 into culture supernatants in high glucose conditions but not in normal glucose following TNF-α treatment (Fig. 4e). HMGB1, an endogenous nuclear protein, is released from cells only when undergoing necrosis^5,6,30^.

### The hyperglycemic shift from apoptosis to necroptosis is driven by RIP1

**Fig. 5:**
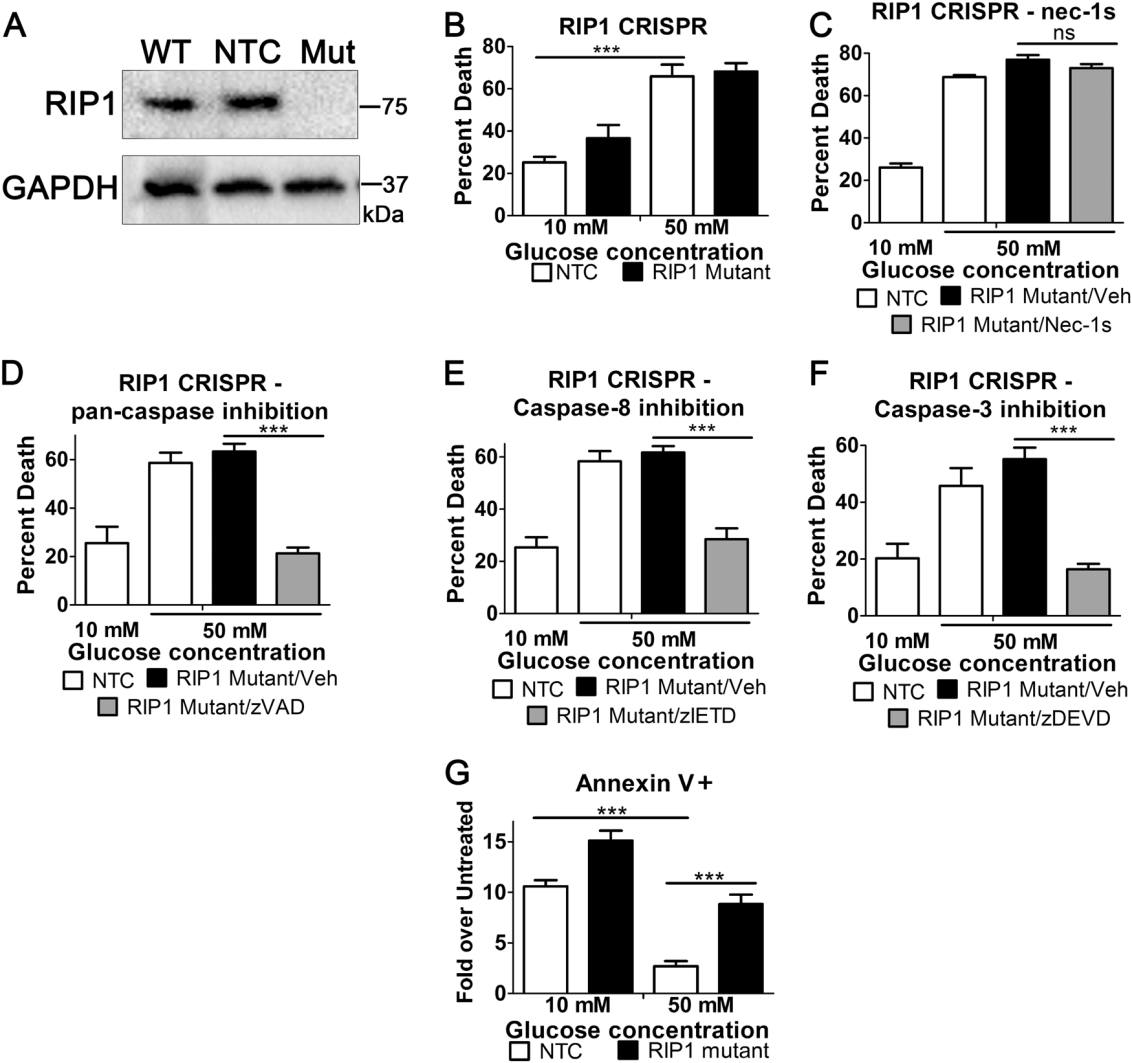
High glucose-enhanced cell death reverts to caspase-dependent death in the absence of RIP1. **a** RIP1 was mutated in U937 cells using CRISPR–Cas9. Mutant cells (Mut) do not express RIP1 protein compared to wild-type (WT) and cells exposed to a non-targeting CRISPR control (NTC). **b** Mutation of RIP1 does not prevent the enhancement of TNF/CHX-induced cell death in high glucose conditions. **c** High glucose-enhanced death of RIP1 mutant cells treated with TNF-α/CHX is not prevented by the RIP1 inhibitor, nec-1s. High glucose-enhanced death of RIP1 mutant cells treated with TNF-α/CHX is prevented by **d** the pan-caspase inhibitor, zVAD, **e** the caspase-8 inhibitor, zIETD, and **f** the caspase-3 inhibitor, zDEVD. **g** Flow cytometry of RIP mutant cells and non-targeting control (NTC) following activation of TNF/CHX-induced apoptosis. Annexin V staining (apoptosis marker) of NTC cells decreases in high glucose following TNF/CHX treatment. Annexin V staining of RIP1 mutant cells does not decrease in high glucose. Two-way ANOVA, *p < 0.05, ***p < 0.001

To further characterize the role of RIP1 in the hyperglycemic shift to necroptosis, we mutated the RIP1 gene in U937 cells using the CRISPR–Cas9 system such that it was not expressed (Fig. 5a). Following this, we conducted death assays to evaluate the effect of the RIP1 mutation on the dynamics of cell death in hyperglycemia. The loss of RIP1 did not prevent increased cell death following induction of apoptosis at high glucose (Fig. 5b) in contrast to results seen with nec-1s (Fig. 3a–c). To rule out any potential off-target effects of the RIP1 inhibitor, cell death was measured in the presence of nec-1s which had no effect on the increased death of RIP1 mutant cells in high glucose (Fig. 5c). We then wondered if cell death in high glucose reverts to caspase-dependent apoptosis in the absence of RIP1. We found that the increased cell death following induction of apoptosis at high glucose was prevented by the pan-caspase inhibitor, zVAD-fmk (Fig. 5d). This suggests a reversion to apoptosis in high glucose conditions in the absence of RIP1 expression. To explore the involvement of individual caspases, we used specific inhibitors of caspase-8 (initiator caspase of extrinsic apoptosis) and caspase-3 (central executioner caspase). Inhibition of these caspases prevented TNF-induced cell death in RIP1 mutant cells in high glucose conditions (Fig. 5e, f). Moreover, the decrease in the apoptosis marker of externalized phosphatidylserine (via annexin V staining) seen in high glucose did not occur in RIP1 mutant cells (Fig. 5g). Overall, these results indicate that cessation of RIP1 expression causes cell death to revert back to caspase-dependent apoptosis in high glucose conditions. This suggests that the expression and presence of RIP1 is critical for the hyperglycemic shift from apoptosis to necroptosis.

### The hyperglycemic shift of apoptosis to necroptosis depends on glycolysis

**Fig. 6:**
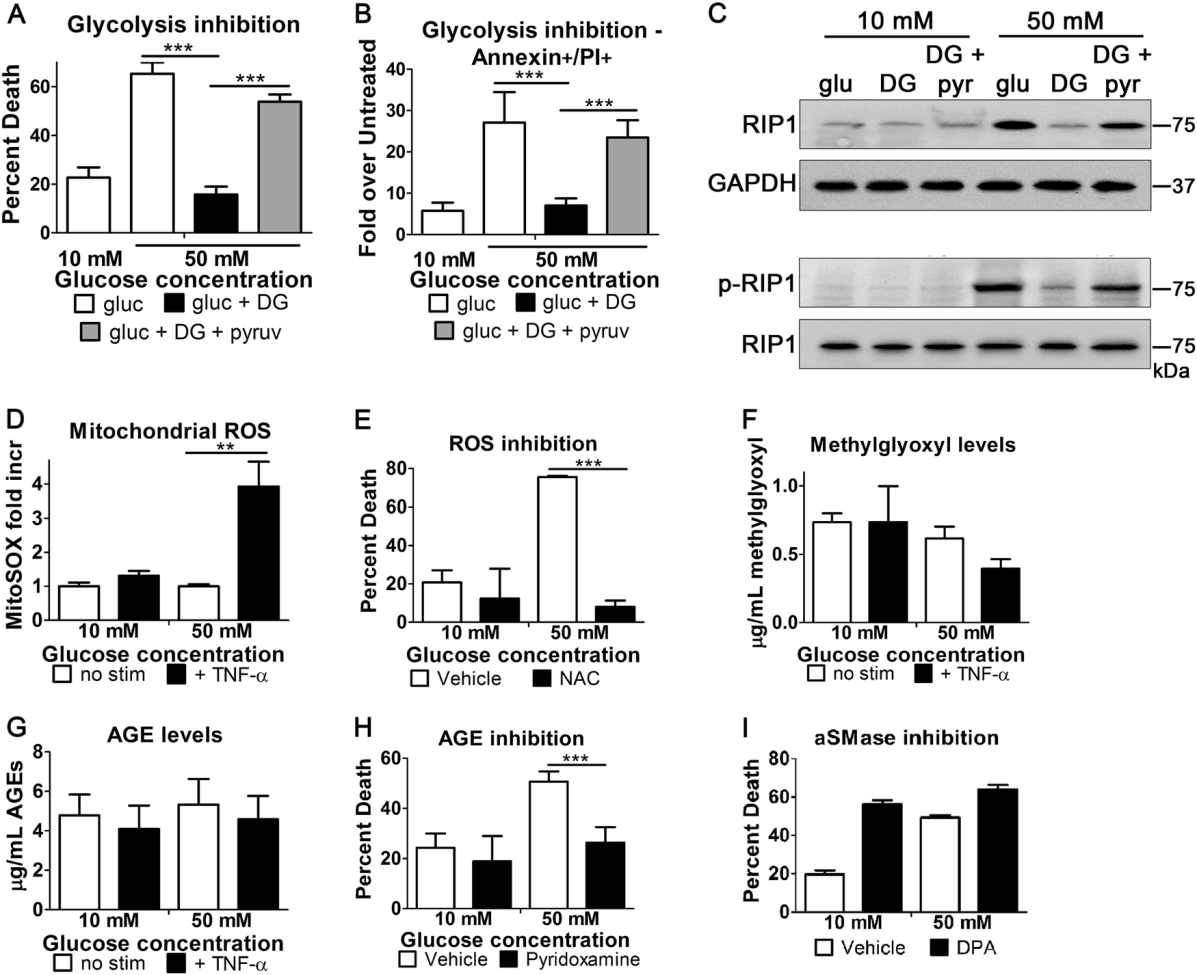
The hyperglycemic shift from apoptosis to necroptosis depends on cellular metabolism and ROS. **a** Hyperglycemic enhancement of TNF/CHX-induced death of U937 cells is prevented by inhibition of glycolysis with 2-deoxyglucose (DG). Addition of exogenous pyruvate (pyruv) restores cell death in those cells that received 2-deoxyglucose. **b** Annexin/PI staining and flow cytometry of U937 cells undergoing TNF/CHX-induced death at 10 and 50 mM glucose. The increase in annexin/PI staining at 50 mM glucose is prevented by 2-deoxyglucose and restored by pyruvate. Two-way ANOVA, ***p < 0.001. **c** High glucose causes an increase in total protein levels and phosphorylation levels of RIP1 following TNF/CHX treatment. Both total levels of RIP1 and phosphorylation levels (p-RIP1) are prevented by inhibition of glycolysis with 2-deoxyglucose. Addition of exogenous pyruvate (pyr) restores protein and phosphorylation levels of RIP1. **d** Flow cytometry of mitoSOX staining in U937 cells stimulated to undergo TNF/CHX-induced death. MitoSOX staining is greatly increased in high glucose conditions (50 mM). **e** Hyperglycemic shift to necroptosis is prevented by the antioxidant, *N*-acetylcysteine (NAC). **f**, **g** ELISAs measuring levels of methylglyoxal (involved in AGE formation) and total AGEs in U937 cells stimulated to undergo TNF/CHX-induced death. Levels of methylglyoxal and AGEs do not change in high glucose. **h** Hyperglycemic shift to necroptosis is partially prevented by inhibition of AGEs with pyridoxamine. **i** The hyperglycemic shift to necroptosis is not affected by inhibition of acid sphingomyelinase with desipramine (DPA). Two-way ANOVA, **p < 0.01, ***p < 0.001

As established in Fig. 1, osmotic effects were not responsible for the hyperglycemic shift to necroptosis. This suggests that glucose metabolism may play a role in the hyperglycemic shift to necroptosis. To determine the role of glucose metabolism, non-metabolizable 2-deoxyglucose (DG) was used as an inhibitor of glycolysis. Cell death following induction of apoptosis was prevented in the presence of DG in high glucose conditions and was restored by subsequent addition of exogenous pyruvate (Fig. 6a). As pyruvate is the end product of glycolysis, this suggests that events downstream of glycolysis are responsible in promoting the hyperglycemic shift to necroptosis. Additionally, annexin/PI staining, a marker of necrosis, increased in high glucose conditions in a glycolysis-dependent manner (Fig. 6b). The annexin/PI staining that was reduced by DG in high glucose conditions was restored with the addition of exogenous pyruvate (Fig. 6b). Next, we analyzed the effect of glycolysis on RIP1 levels, shown to be critical for the hyperglycemic shift to necroptosis (Fig. 5). Total levels of RIP1 increased in high glucose conditions but was prevented by glycolysis inhibition with DG (Fig. 6c). The subsequent addition of exogenous pyruvate restored levels of RIP1 (Fig. 6c). After normalizing total levels of RIP1, we determined that the phosphorylation of RIP1 also increased in high glucose conditions (Fig. 6c). Moreover, the levels of phosphorylated RIP1 decreased following addition of DG and were restored by the subsequent addition of exogenous pyruvate (Fig. 6c).

### The hyperglycemic shift of apoptosis to necroptosis depends on mitochondrial ROS

Glycolysis and downstream metabolism leads to production of toxic byproducts, such as ROS and AGEs^23,24^. Since both ROS and AGEs are downstream effectors of necroptosis^24^, we investigated the contribution of these toxic species in the hyperglycemic shift to necroptosis. First, we measured mitochondrial ROS and observed that ROS increased markedly in high glucose conditions following TNF-α treatment (Fig. 6d). We then measured cell death in the presence of the antioxidant, *N-*acetylcysteine (NAC), and found that inhibition of ROS prevented the shift to necroptosis in high glucose conditions (Fig. 6e). Next, we determined the involvement of AGEs by measuring the levels of the AGE precursor, methylglyoxal (MG) as well as total AGEs. Both MG and AGE levels did not change in high glucose following TNF-α treatment (Fig. 6f, g). Inhibition of AGEs with pyridoxamine blunted necroptotic cell death at high glucose (Fig. 6h), suggesting that AGEs may have a partial role in the hyperglycemic shift from apoptosis to necroptosis. However, it is known that pyridoxamine may also inhibit ROS^31–33^ which provides an alternative interpretation for the results in Fig. 6h. Acid sphingomyelinase (aSMase) creates ceramide which is another downstream effector of necroptosis^5,34^. However, unlike ROS and AGEs, this effector does not depend on glycolysis^5,34^. Inhibition of aSMase had no effect on the high glucose enhancement of cell death following induction of apoptosis (Fig. 6i). Therefore, it can be concluded that glycolysis and its downstream byproducts, particularly ROS, have a role in mediating the shift from apoptosis to necroptosis in hyperglycemic conditions.

### Hyperglycemic conditions induce a shift from apoptosis to necroptosis in primary human T cells

**Fig. 7:**
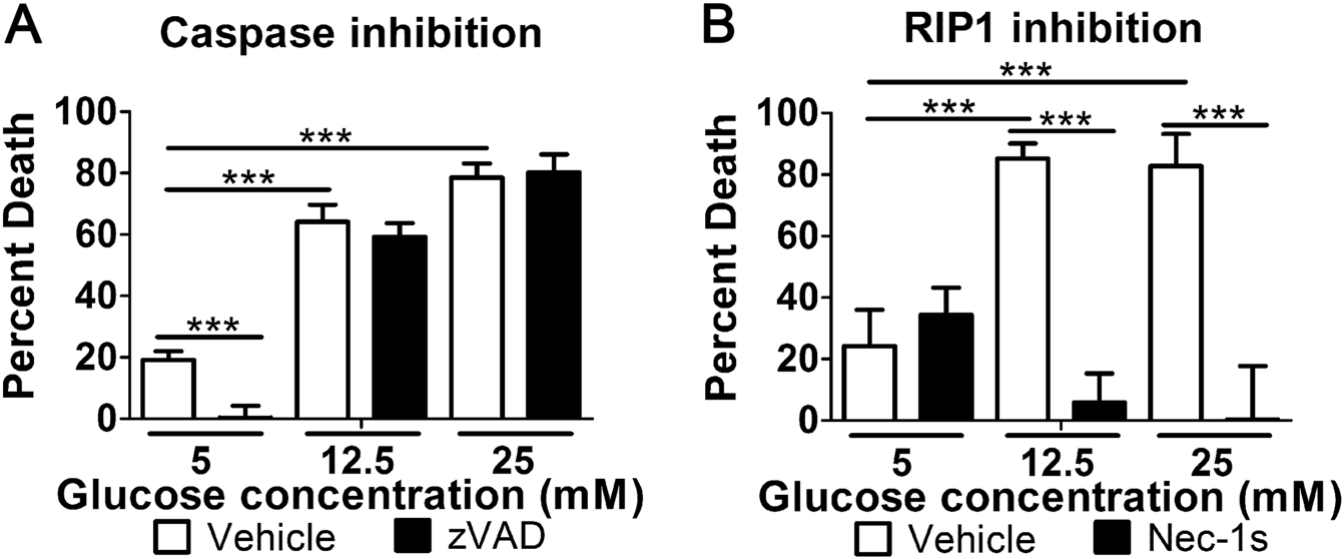
High glucose shifts extrinsic apoptosis to necroptosis in primary human T cells. **a** Levels of TNF-induced death of primary human T cells increase in high glucose (12.5 mM, 25 mM) relative to normal glucose (5 mM). Primary T cell death is prevented by the pan-caspase inhibitor, zVAD, at 5 mM but not at 12.5 or 25 mM glucose. **b** Enhanced death of primary T cells in hyperglycemic conditions (12.5 mM, 25 mM) is prevented by inhibition of RIP1 with nec-1s. Two-way ANOVA, ***p < 0.001

We wanted to determine if the hyperglycemic shift from extrinsic apoptosis to necroptosis occurred in primary cells. For this, we used primary human T cells and measured TNF-induced cell death in normal and high glucose conditions. Since the normal concentration of glucose in serum is 5 mM, we used 12.5 and 25 mM glucose as high glucose conditions. For these experiments, CHX was not included. High glucose led to enhanced death of primary T cells following induction of apoptosis (Fig. 7). Pan-caspase inhibition with zVAD-fmk prevented TNF-induced cell death in normal glucose but did not prevent the cell death seen in high glucose conditions (Fig. 7a). Conversely, inhibition of RIP1 with nec-1s did not prevent TNF-induced cell death at normal glucose but inhibited it significantly in high glucose conditions (Fig. 7b). Importantly, these results establish that the hyperglycemic shift from apoptosis to necroptosis occurs in primary human cells and is not only a phenomenon of cell lines.

### Hyperglycemia causes an increase in levels of necroptosis kinases concurrent with a decrease in executioner caspases and PARP1 in neonatal mice following HI brain injury

**Fig. 8:**
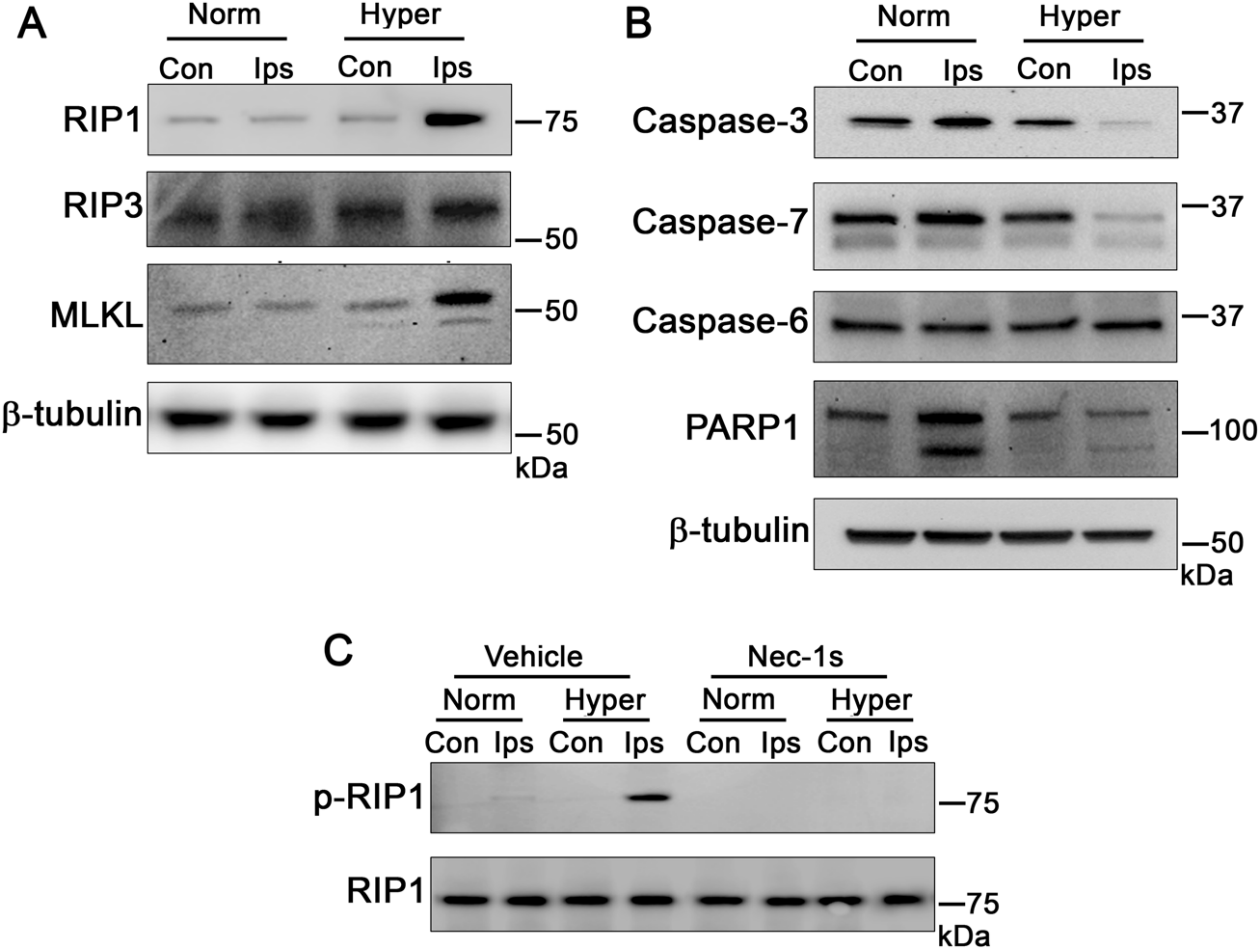
Levels and activity of necroptosis kinases increase in cerebral tissue while caspase levels and PARP1 cleavage decrease during hyperglycemia and neonatal hypoxia-ischemia (HI) brain injury *in vivo*. Western blots from ipsilateral (ips) and contralateral (con) cerebral tissue samples taken from neonatal mice with normal glucose levels (norm) or hyperglycemia (hyper) following HI-brain injury. **a** Total levels of RIP1 and MLKL increase in ipsilateral tissue of hyperglycemic mice that received HI-brain injury. Total levels of RIP3 remain unchanged. **b** Levels of caspase-3 and caspase-7 decrease in ipsilateral tissue of hyperglycemic mice that received HI-brain injury while caspase-6 remains unchanged. PARP1 cleavage is observed in ipsilateral tissue of normal mice and decreases in hyperglycemic mice. **c** Phosphorylation of RIP1 (p-RIP1) increases in ipsilateral tissue from hyperglycemic mice that received HI-brain injury and is prevented by the RIP1 inhibitor, nec-1s

Our previous work established exacerbation of cerebral HI injury in hyperglycemic neonatal mice in a RIP1-dependent manner^23^. Based on our current work, we hypothesized that this may be the result of a shift from apoptosis to necroptosis *in vivo*. To determine the translational relevance of the hyperglycemic shift from apoptosis to necroptosis in neonatal HI-brain injury, we determined the levels of RIP1, RIP3, MLKL, executioner caspases, and PARP1 in cerebral tissue samples from normal or hyperglycemic neonatal mice subjected to HI-brain injury. We observed an increase in the total levels of RIP1 and MLKL in ipsilateral tissue from hyperglycemic mice that received the injury compared to euglycemic littermates (Fig. 8a). There was no detectable change in levels of RIP3 (Fig. 8a). Moreover, levels of caspase-3 and -7 decreased in the ipsilateral tissue of hyperglycemic mice subjected to HI-brain injury (Fig. 8b). Levels of caspase-6 remained unchanged (Fig. 8b). Moreover, PARP1 cleavage was prominent in euglycemic mice but decreased in hyperglycemic mice (Fig. 8b). Uncleaved levels of PARP1 also decreased in hyperglycemic mice (Fig. 8b) which is a marker of necroptosis^35^. We also saw an increase in levels of phosphorylated RIP1 in the ipsilateral tissue of hyperglycemic mice, which was prevented by nec-1s (Fig. 8c). Overall, these *in vivo* results are consistent with our *in vitro* observations and suggest that the hyperglycemic shift from apoptosis to necroptosis participates in the exacerbation of neonatal HI-brain injury.

## Discussion

### A novel shift in cell death mechanisms due to high glucose

In the present study we have demonstrated a novel shift from caspase-dependent extrinsic apoptosis to RIP1-dependent necroptosis in high glucose conditions. This is distinct from our previous work in which we showed hyperglycemic enhancement of cell death in response to specific stimuli of necroptosis^23^. In those experiments necroptosis was induced in normal and hyperglycemic conditions via artificial inhibition of apoptosis and was shown to increase in hyperglycemic conditions^23^. In contrast to that research, we currently show that hyperglycemic conditions produce an alteration in cell death mechanisms. Specifically, necroptosis is activated in high glucose, despite the fact that cell death in response to the same stimuli proceeds via apoptosis in euglycemic conditions. This shows that hyperglycemia is capable of causing the endogenous inhibition of apoptosis, shifting the balance of cell death to necroptosis.

We have shown that this shift from apoptosis to necroptosis occurs in multiple cell types including primary human T cells in response to two different extrinsic stimuli (TNF-α and FasL). This is significant as there is overlap between the initiation of extrinsic apoptosis and necroptosis^5,14,36^. As a result of this overlap, these pathways are in constant competition^37^. RIP1, RIP3, and other molecular switches maintain the balance of PCD^37^, however, situations that result in one form of PCD prevailing over the other are not well understood. We now identify a high glucose environment as a specific condition that shifts the balance of cell death toward the necroptosis module, shedding light on mechanisms that govern the balance between these pathways. Such a shift is not trivial as it represents a shift from a non-inflammatory to an inflammatory form of cell death^38–40^. This shift in cell death depends on glucose metabolism (Fig. 6a–c) and is not the result of osmotic effects (Fig. 1). Additionally, we have demonstrated that it involves a high degree of ROS (Fig. 6d, e), a toxic byproduct of cellular metabolism^41^. That this occurs specifically in response to high glucose suggests the shift from apoptosis to necroptosis is linked to hyperglycemia.

We have chosen to classify this as a hyperglycemic shift from apoptosis specifically to necroptosis for several reasons. First, increasing levels of glucose resulted in a conversion from caspase-dependent to RIP1-dependent cell death (Figs. 2 and 3). In addition, we observed a decrease in the abundance and activity of executioner caspases-3, -6, and -7 (Fig. 2e–h), critical for apoptosis^36^, with a concomitant increase in the abundance and activity of RIP1 (Fig. 6c), the initiator kinase of necroptosis^5,14^. Additionally, we observed increases in RIP3 phosphorylation and membrane translocation of MLKL, both critical events in necroptosis^5,14,22^, during the hyperglycemic shift to necroptosis (Fig. 4a, b). Finally, cells undergoing TNF-induced death in hyperglycemic conditions display necrotic characteristics with a decrease in apoptotic characteristics: (1) membrane permeability increased while phosphatidylserine externalization decreased (Fig. 4c) and (2) cell size initially expanded followed by a sharp decrease in size as opposed to a steady decrease in cell size in normal glucose conditions (Figs. 4d and 3). Nuclear protein HMGB1 was released from dying cells only in hyperglycemic conditions (Fig. 4e).

### Critical role for RIP1 in hyperglycemic shift to necroptosis

Our results indicate that RIP1 expression is critical for the hyperglycemic shift from apoptosis to necroptosis and reveal additional information about the balance between these two pathways. Using CRISPR–Cas9, we mutated RIP1 and abolished its expression (Fig. 5a). Unexpectedly, this resulted in no inhibition of the increased cell death seen in hyperglycemic conditions (Fig. 5b). Interestingly, this revealed that in the absence of RIP1 expression, caspase-dependent apoptosis substitutes for RIP1-dependent necroptosis in high glucose (Fig. 5d–g). This underscores a critical role for RIP1 in the hyperglycemic shift to necroptosis as it does not occur in its absence. In addition, this reveals an important backup role for apoptosis in situations of hyperglycemia where RIP1 is absent.

### Translational relevance of a shift from apoptosis to necroptosis during hyperglycemia

As necroptosis is highly inflammatory and more damaging than apoptosis^38,39^, a shift to necroptosis may have physiological relevance to injuries that are exacerbated in hyperglycemic individuals. In fact, our results indicate relevance of this phenomenon to the exacerbation of neonatal HI-brain injury during hyperglycemia *in vivo* (Fig. 8). Previously, we showed that cerebral damage due to neonatal HI-brain injury was exacerbated during hyperglycemia in a RIP1-dependent manner^23^. Our work here suggests that this RIP1-dependent exacerbation is indeed the result of a hyperglycemic shift from apoptosis to necroptosis *in vivo*. This is due to the observation that levels of RIP1, phosphorylated RIP1, and MLKL increase in cerebral tissue from hyperglycemic mice subjected to HI-brain injury while levels of caspases-3 and -7 decrease (Fig. 8). Moreover, PARP1 cleavage^42^ was observed in euglycemic mice indicating activation of apoptosis while its cleavage decreased in hyperglycemic mice that received HI-brain injury (Fig. 8b). In addition, uncleaved PARP1 decreased, which serves as a marker for the activation of necroptosis^35^ (Fig. 8b). The prevalence of necroptosis kinases over caspases during neonatal HI-brain injury in the context of hyperglycemia indeed suggests that necroptosis predominates over apoptosis in this situation. Overall, this demonstrates that mode of PCD in neonatal HI-brain injury is dynamic and responsive to changes in cellular glucose. Once again, this may provide an explanation at the cellular/molecular level for the propensity of hyperglycemia to exacerbate this and other ischemic injuries.

## Materials and methods

### Cell culture

U937 monocytes and Jurkat T cells were obtained from ATCC (Manassas, VA, USA). The U937 and Jurkat cells were cultured in RPMI 1640 medium with 10% fetal bovine serum (FBS). HEK293T cells were kindly provided by Dr. H. John Sharifi (ACPHS) and were cultured in Dulbecco’s Modified Eagle’s Medium (DMEM) supplemented with 10% FBS. Primary lymphocytes were purchased from the Elutriation Core Facility at University of Nebraska Medical Center. The lymphocytes were cultured with RPMI 1640 supplemented with 5 mM glucose and 10% FBS and were differentiated into T cells with ImmunoCult Human CD3/CD28 T Cell Activator and human IL-2 as per manufacturer’s instructions (Stemcell Technologies Inc.). All cells were cultured at 37 °C and 5% CO_2_.

### Cell death assays

Cells were first incubated in RPMI 1640 media containing designated amounts of glucose, mannitol, or sucrose for 24 h at 37 °C and 5% CO_2_. Cells were then washed and resuspended in RPMI 1640 medium containing 10 mM glucose. Following this cells were treated with cell death stimuli for 24 h at 37 °C and 5% CO_2_ after which WST-1 reagent was used to measure cell death according to the manufacturer’s instructions (Clontech Laboratories, Inc.). Unless indicated otherwise, cells were treated with 0.4 lytic units (LD_50_) of TNF-α or FasL and 0.6 lytic units (LD_50_) of staurosporine. 1 lytic unit/LD_50_ is defined as the dose of cell death stimulus that results in 50% cell death as described previously^23^. Aside from experiments using primary human T cells, in all cases, 0.25 µg/mL CHX was administered with cell death stimuli for 24 h at 37 °C and 5% CO_2_. Pharmacologic inhibitors were incubated with cells at 37 °C and 5% CO_2_ for 1 h prior to addition of cell death stimuli. The inhibitors were used at the following concentrations: zVAD-fmk (50 µM), Nec-1s (50 µM), zDEVD-fmk (30 µM), zIETD-fmk (30 µM), zLEHD-fmk (10 µM), desipramine (20 µM), *N*-acetylcysteine (10 mM), and pyridoxamine (1 mM). Using WST-1, percent viability was calculated as follows: (abs cell death stimulus + CHX)/(abs neg + CHX)*100.

### CRISPR/Cas9-mediated RIP1 mutation

RIP1 knockout U937 cell lines were generated using the CRISPR–Cas9 lentiviral system. All-in-one lentiviral expression vectors containing a single guide RNA (gRNA) and Cas9 targeting RIP1 were obtained from TransOMIC Technologies (Cat. # CAHS1001-8737). Lentiviral packaging plasmids (pLP1, pLP2, and pLP/VSVG) were obtained from Thermo Fisher Scientific. HEK293T cells were transfected with 1.5 µg of pLP1, 1.5 µg of pLP2, 1.5 µg of pLP/VSVG, and 1.5 µg of non-targeting control vector or CRISPR–Cas9 lentiviral vector using the X-tremeGENE 9 DNA Transfection Reagent (Roche). After 48 h of transfection, lentiviral supernatants were collected by centrifugation and used to spinoculate U937 cells. To spinoculate, U937 cells were centrifuged with the lentivirus at 1200 rpm for 1 h and then rotated for an additional hour. After spinoculation, the cells were incubated overnight at 37 °C and 5% CO_2_ and cultured with 3 µg/mL puromycin for 2 weeks. Single cell clones were then obtained by limiting dilution in 96-well plate and subsequently expanded when the cells reached high confluence. The knockout of RIP1 in U937 cells were evaluated using western blot and functional analysis.

### Preparation of cell lysates

2 × 10^7^ U937 cells were treated with 25 ng/mL TNF-α and 0.25 µg/mL CHX for 2.5 h at 37 °C and 5% CO_2_. Cells were then lysed with Tris-buffered saline (TBS) supplemented with 1% Triton X-100 and 1× Halt protease inhibitor (Thermo Scientific) for 30 min on ice. Clear lysate was obtained by centrifugation at 14,000 rpm for 15 min at 4 °C. For analysis of brain tissue, samples from mice were homogenized in RIPA buffer and sonicated for 3 × 20 s at 20% pulse. The samples were then incubated on ice for 30 min and centrifuged at 14,000 × *g* for 15 min at 4 °C to pellet cell debris.

### Immunoprecipitations

Immunoprecipitation of HMGB1 was performed in culture supernatants. U937 cells were incubated in 10 or 50 mM glucose overnight at 37 °C and 5% CO_2_. Cells were washed and treated with 20 ng/mL TNF-α overnight. Cells were centrifuged and supernatant was isolated. 10 µg of anti-human HMGB1 (Cell Signaling Technology) was added to supernatants and allowed to incubate with gentle mixing overnight at 4 °C. Supernatants were then incubated with Protein G Plus agarose beads (Pierce) for 2 h at room temperature. Beads were washed, resuspended in 1× Laemmli buffer, run on SDS-PAGE, and western blotted. Immunoprecipitation of RIP1 from lysates prepared from tissue homogenates was performed by addition of 10 µg of anti-mouse RIP1 (Cell Signaling Technology) followed by procedure described above.

### Cell fractionation

U937 cells were grown at 37 °C and 5% CO_2_ overnight in RPMI 1640 media containing indicated levels of glucose. After overnight incubation, cells were adjusted to 1 × 10^6^ cells/ml and suspended in 10 mL RPMI with normal glucose levels. CHX and TNF-α were added to a final concentration of 0.25 µg/mL and 25 ng/mL, respectively. Cells were incubated for 2.5 h, pelleted and washed with ice cold PBS. Cells were fractionated using the Cell Fractionation Kit (Cell Signaling Technology) according to manufacturer’s instructions.

### Western blots

Lysates, fractionation samples, immunoprecipitates, and tissue homogenates were run on SDS-PAGE and transferred to a PVDF membrane and blocked in TBS-T buffer with 5% milk for 30 min at room temperature. The blots were then incubated with diluted primary antibody in TBS-T buffer with 5% milk overnight at 4 °C. All primary antibodies were obtained from Cell Signaling Technology, unless otherwise indicated. Primary antibodies were used at the following dilutions: anti-human MLKL (1:500), anti-human CD71 (1:1500), anti-human GAPDH (1:5000), anti-human caspase-8 (1:1000), anti-human caspase-3 (1:1000), anti-human/mouse caspase-6 (1:1000), anti-human/mouse caspase-7 (1:1000), anti-human HMGB1 (1:1000), anti-human RIP1 (1:1000), anti-human p-RIP1 (1:1000), anti-mouse RIP1 (1:1000), anti-phospho S/T (1:500), anti-human RIP3 (1:1000), anti-human p-RIP3 (1:1000), anti-mouse caspase-3 (1:1000), anti-mouse PARP1 (1:1000), anti-mouse RIP3 (1:500), and anti-mouse MLKL (EMD Millipore, 1:1000). After washing with TBS-T with 5% milk, the blots were incubated with secondary HRP-conjugate antibodies for 1 h at room temperature. Blots were developed by chemiluminescence and read in a Bio-Rad ChemiDoc XRS+.

### Flow cytometry (Annexin/PI, MitoSox, mitochondria levels, FLICA)

For flow cytometry analyses 10,000 events were collected for each sample after gating out debris. Sample data were collected utilizing a BD FACSVerse flow cytometer. Data files were analyzed using FlowJo V10. Prior to analysis, U937 cells were incubated in 10 or 50 mM glucose for 24 h at 37 °C and 5% CO_2_. Cells were washed and treated with 25 ng/mL TNF-α at 37 °C and 5% CO_2_ for 6 h. For mitochondrial ROS, MitoSOX superoxide indicator or MitoTracker (Invitrogen) was added to cells to a final concentration of 5 μM. For Annexin/PI, the eBiosciences Annexin V apoptosis kit (Invitrogen) was used according to the manufacturer’s instructions. For caspase activity, the Vybrant FAM FLICA kit (Molecular Probes) was used according to the manufacturer’s instructions.

### Cell diameter measurements

U937 cells were incubated in 10 or 50 mM glucose overnight and then treated with 25 ng/mL TNF-α for different time points up to 5 h. At each time point, cellular diameter was measured using a Countess II cell counter (Life Technologies).

### Measurement of AGE and MG levels

For measurement of AGEs, U937 cells were incubated in 10 or 50 mM glucose for 24 h at 37 °C and 5% CO_2_. Cells were washed and treated with 25 ng/mL TNF-α at 37 °C and 5% CO_2_ for 6 h. Cells were then lysed and AGEs or MG were measured using the Oxiselect AGE and methylglyoxal competitive ELISA kits (Cell Biolabs) according to the manufacturer’s instructions.

### *In vivo* brain HI model

Hyperglycemia was induced in neonatal (p10) mice subjected to regional HI-brain injury according to our description in ref. ^23^ All studies were conducted according to a protocol approved by the Columbia University Institutional Animal Care and Use Committee (IACUC) and in accordance with the Association for Assessment and Accreditation of Laboratory Animal Care guidelines.

### Statistical analysis

Two-way ANOVA with Bonferroni posttest was used to analyze used to analyze significance of all the data using GraphPad Prism 5.0.

## Electronic supplementary material

Figure S1

Figure S2

Figure S3

Supplementary Information
