# Supplementary figures and images for: Hyperglycemia potentiates a shift from apoptosis to RIP1-dependent necroptosis
Source: Cell Death Discov. 2018 May 10;4:55. doi: 10.1038/s41420-018-0058-1 (PMC5945624; doi:10.1038/s41420-018-0058-1)

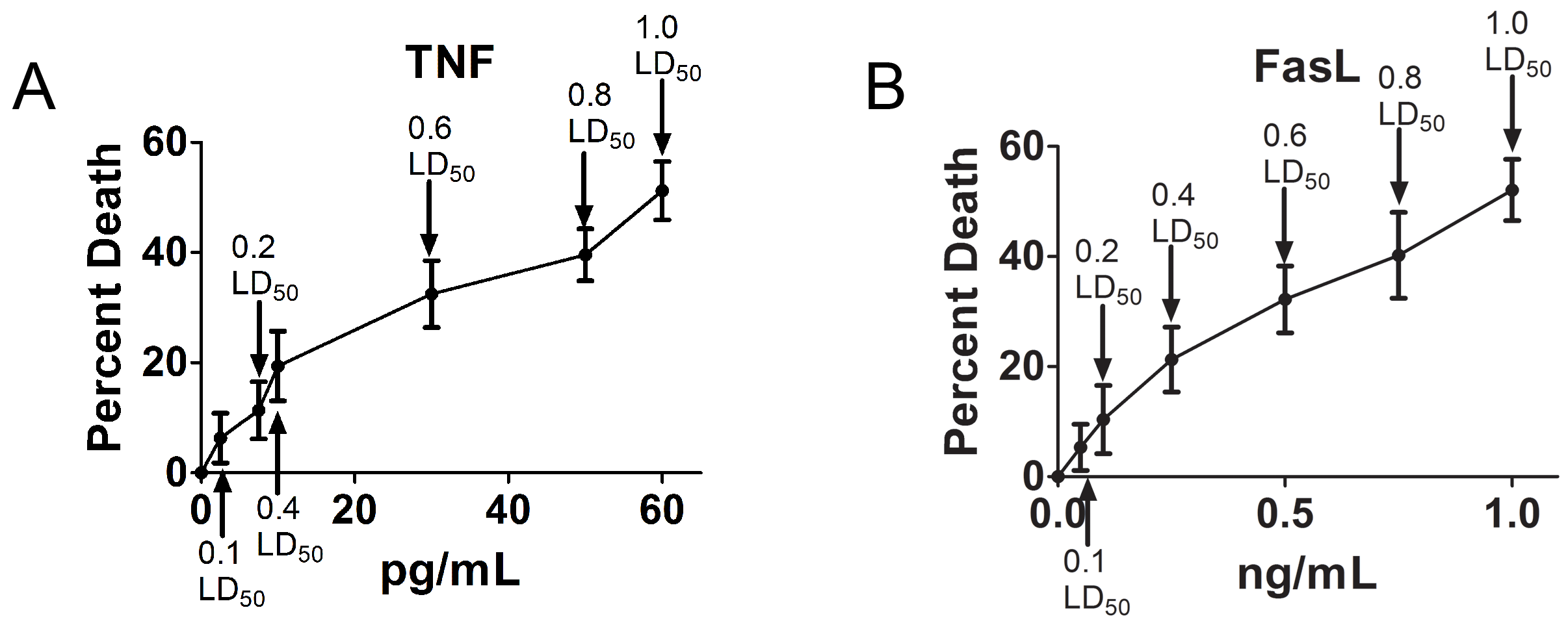

Supplement: Supplementary file 2 — Figure S2 [file 41420_2018_58_MOESM2_ESM.tif]

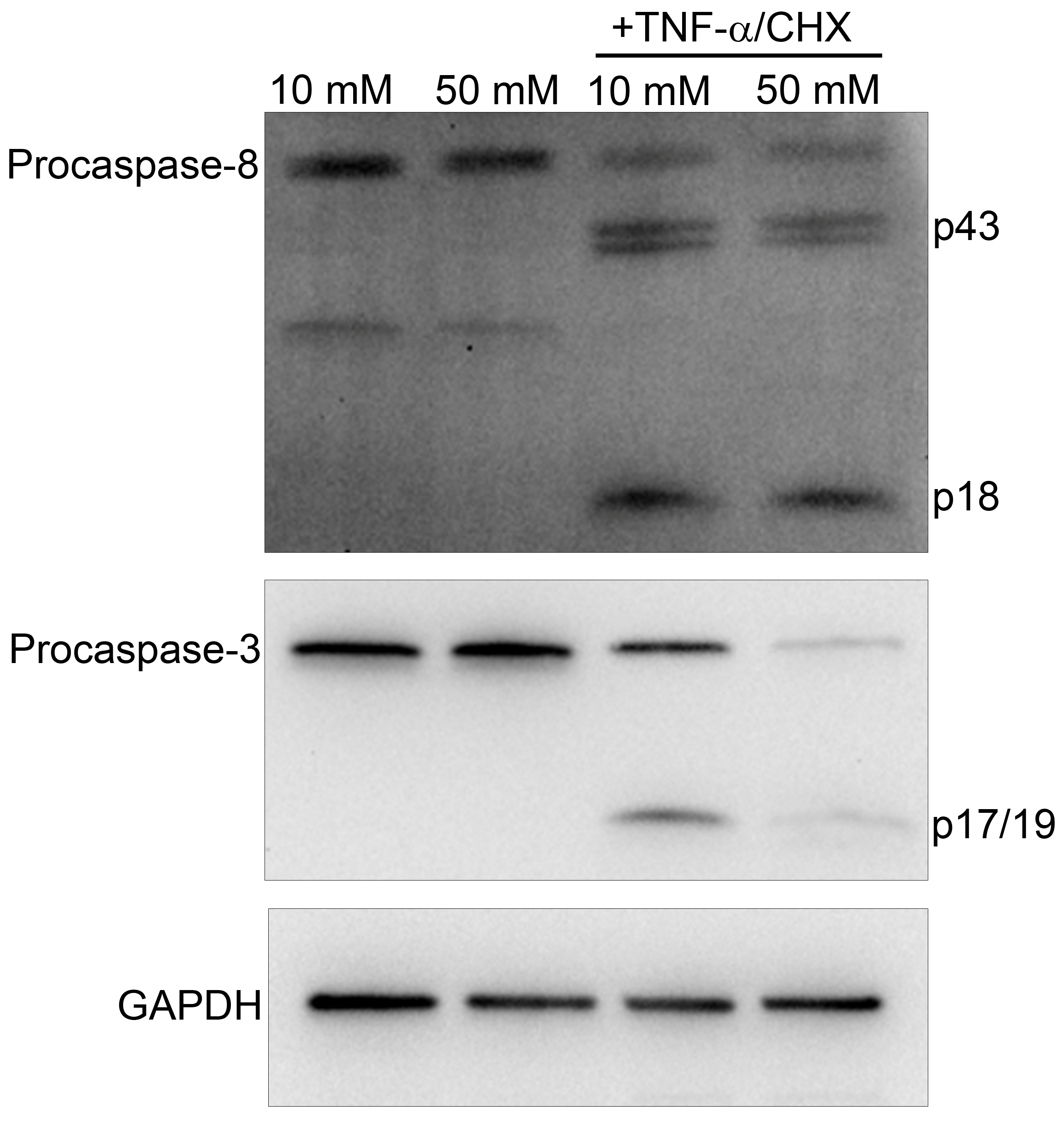

Supplement: Supplementary file 3 — Figure S3 [file 41420_2018_58_MOESM3_ESM.tif]
