# Supplementary material for: Hyperglycemia potentiates a shift from apoptosis to RIP1-dependent necroptosis
Source: Cell Death Discov. 2018 May 10;4:55. doi: 10.1038/s41420-018-0058-1 (PMC5945624; doi:10.1038/s41420-018-0058-1)
Supplement: Supplementary file 4 — Supplementary Information [file 41420_2018_58_MOESM4_ESM.docx]

Supplemental Figures

**Figure S1: 250 ng/mL CHX causes minimal cell death of U937 monocytes in the absence of TNF-α.** U937 monocytes cultured in 10 or 50 mM glucose were treated with 250 ng/mL CHX or a combination of 250 ng/mL CHX + TNF-α for 24 h. CHX alone causes minimal cell death at 10 mM glucose which is not affected at 50 mM glucose. Cell death induced by CHX + TNF-α is greatly enhanced in 50 mM glucose.

**Figure S2: Different doses of TNF-α and FasL correspond to different lytic units.** U937 monocytes or Jurkat T cells cultured in 10 mM glucose were treated with different doses of **A.)** TNF-α/CHX or **B.)** FasL/CHX for 24 h, respectively. Each cell death stimulus causes a dose-dependent increase in cell death. Lytic units (LD_50_) are pointed out for each dose. The definition of 1 lytic unit (LD_50_) is the dose of cell death stimulus which produces 50% cell death.

**Figure S3: Full-length procaspase-8 decreases in abundance in U937 monocytes treated with TNF-α/CHX.** Western blots of lysates from untreated U937 monocytes or those treated with TNF-α/CHX. Prior to treatment, cells were cultured in 10 or 50 mM glucose. In contrast to procaspase-3, procaspase-8 decreases in abundance following treatment with TNF-/CHX.
